# 'A perfect storm' or missed care? Focus group interviews with dementia care professionals on Advance Care Planning

**DOI:** 10.1186/s12877-023-04033-7

**Published:** 2023-05-21

**Authors:** Annika Tetrault, Maj-Helen Nyback, Lisbeth Fagerström, Heli Vaartio-Rajalin

**Affiliations:** 1 Department of Caring Science, Faculty of Education and Welfare Studies, Åbo Akademi University, Strandgatan 2, 65100 Vaasa, Finland; 2 The Wellbeing Services County of Ostrobothnia, Sandviksgatan 2-4, Vasa, 65100 Finland; 3 Novia University of Applied Sciences, Wolffska Vägen 31, 65200 Vaasa, Finland; 4 Faculty of Health and Social Sciences, University of South-Eastern Norway, PO 235, 3603 Kongsberg, Norway

**Keywords:** Dementia, Advance care planning, Missed care

## Abstract

**Background:**

Dementia is one of the leading causes of dependency and disability among older people and currently the seventh leading cause of death among all diseases. In recent years, healthcare research in Advance Care Planning in dementia care has received increased attention. Advance Care Planning is a discussion process conducted in anticipation of future deterioration of a person’s health condition. The purpose of the study was to investigate the views of dementia nurses and geriatricians on Advance Care Planning in dementia care.

**Methods:**

The study design is a qualitative study using semi-structured focus group interviews with dementia care professionals in a region in Western Finland. A total of seventeen dementia care professionals participated. A modified version of the Qualitative Analysis Guide of Leuven was used for the data analysis.

**Results:**

The data analysis identified one main theme and three sub-themes describing the views of dementia nurses and geriatricians on Advance Care Planning in dementia care. The main theme was the ‘perfect storm’ with sub-themes relating to the person with dementia, the care process, and the care professional. The unfavorable circumstances creating a ‘perfect storm’ are related to the nature of the illness and the associated stigma, to the unclarity in the suggested care path with inadequate guidelines for Advance Care Planning, and to the demands placed on dementia nurses and geriatricians, as well as to insufficient resources.

**Conclusions:**

Dementia nurses and geriatricians acknowledge the importance of advance directives and express a generally positive view of Advance Care Planning in dementia care. They also hold views on a number of factors which affect the conditions for conducting Advance Care Planning. The lack of Advance Care Planning in dementia care can be seen as a form of missed care caused by multiple forces coming together simultaneously.

**Supplementary Information:**

The online version contains supplementary material available at 10.1186/s12877-023-04033-7.

## Background

Dementia is a global issue of concern. Worldwide, more than 55 million people live with dementia and the number of people living with dementia is expected to increase to 152 million by 2050 [1]. Every year there are close to 10 million new cases with Alzheimer’s disease which accounts for 60–70% of overall cases. Dementia is one of the leading causes of dependency and disability among older people and is currently the seventh leading cause of death among all diseases. Dementia is usually progressive in nature, with cognitive decline affecting orientation, thinking, memory, comprehension, language, learning capacity, and judgement. Mood and behavior changes, emotional control, and/or motivation commonly accompany or precede deterioration in cognitive function [2].

In recent years, healthcare research in Advance Care Planning (ACP) in dementia care has received increased attention. ACP is a discussion process conducted in anticipation of future deterioration of a person’s health condition [3]. A definition of ACP as proposed by a multi-disciplinary Delphi panel is provided in Table 1 [4].

**Table 1:** Advance Care Planning definition

*Advance care planning enables individuals who have decisional capacity to identify their values, to reflect upon the meanings and consequences of serious illness scenarios, to define goals and preferences for future medical treatment and care, and to discuss these with family and health-care providers. ACP addresses individuals’ concerns across the physical, psychological, social, and spiritual domains. It encourages individuals to identify a personal representative and to record and regularly review any preferences, so that their preferences can be taken into account should they, at some point, be unable to make their own decisions *[4]	

Recent healthcare research in ACP has focused on different areas, including the roles of nurses [5, 6], doctors [6–8], family members [9–12] and the person with dementia [9, 10, 12, 13]. Other studies have focused on the effects and feasibility of different approaches [14, 15] and have described ACP and the dementia approach of different interventions [16]. Factors that facilitate or hinder ACP in dementia care have been explored [17–19] and resulting insights and increased knowledge have led to the creation of guidelines [20, 21] and educational programs [5]. The consensus seems to be that ACP should be initiated as soon as possible [22] as decision-making capacity and illness awareness diminish as the illness progresses [19, 23, 24]. However, one of the questions that remain largely unanswered and challenging concerns the timing of ACP in dementia care as confirmed by a recent narrative review [25]. Additionally, ACP is rarely conducted with dementia patients [26] despite ACP being seen as especially important for people living with dementia as self-determination capacity and abstract thinking ability diminish with illness progression [14, 19, 27]. While research in dementia care ACP has often focused on the advanced stage of illness [28, 29], research involving the person with dementia in the early phase of illness is needed [30].

Finnish laws about patient rights state that healthcare services and care should be arranged together with the patients and that everyone has the right to refuse care [31–33]. In Finland palliative care in general has received increased attention during the last two decades [34–36]. In 2016, the Finnish Ministry of Social Affairs and Health appointed a national group to work on uniform criteria for access to care. Part of that work focused on drafting a proposal for the provision of end-of-life and palliative care. The objective of this proposal is to ensure equal access to palliative and end-of-life care throughout the country [36, 37]. ACP evolved from and includes such concepts as living wills, advance directives, advance decisions to refuse treatment, and lasting power of attorney [38]. These concepts have been in use for a long time in Finland [39], however, ACP as a systematic process in itself has not received attention in Finland until fairly recently [37, 39] and there are few relevant studies in the literature [39]. Several international studies have indicated that nurses and nurse practitioners are well suited to participate in ACP process development and well positioned to initiate and lead ACP conversations [9, 23, 40–43]. Nurses’ experiences of ACP have been explored [6], their understanding of ACP has been examined [44], and their confidence levels and motivation for ACP have been described [45]. A training needs analysis of Admiral Nurses in the United Kingdom showed that the skills and confidence of nurses would benefit from combining communication training with supervised practice, shadowing, and access to materials that facilitate discussion [46]. As ACP in general is not a well-known concept in Finland, studies exploring ACP in the Finnish healthcare context are needed. A study focusing on nurses in Finnish dementia care is relevant for the development of ACP in this context.

## Methods

This study is part of a PhD project, which aims to develop a model for a relationship-centered ACP process in early-phase dementia care. The aim of the current study is to investigate the views of dementia nurses and geriatricians on ACP in dementia care. The objective of the study was kept non-specific to obtain as many viewpoints as possible from the study participants on the many aspects of ACP, including when to initiate the process, whose responsibility it is to take the initiative, and the ethical aspects of conducting or refraining from ACP.

### Design and method of data collection

The approach of the current study is a qualitative study with semi-structured focus group interviews conducted with dementia care professionals. In the last decades, the use of focus group interviews in qualitative health research has been increasing. Focus groups have the potential to provide in-depth information in a relatively short period of time [47, 48]. The participants are selected based on their experience with and/or knowledge of the specific matter at hand [49]. The Standards for Reporting Qualitative Research (SRQR) have been used to report the study [50].

### Context and participants

In the European Dementia Monitor report of 2020, Finland was ranked sixth in care availability, first in care affordability, and as number eight in overall ranking out of the 36 countries and regions evaluated. The Finnish dementia service structure provides a wide range of services and services are available to all [51]. In Finland, memory clinics are often situated within a larger primary care clinic. In memory clinics, registered nurses (RNs) work together with physicians. Memory clinics are easily accessible. Individuals or their family members who suspect the onset of dementia can contact the dementia nurse directly without the referral of a physician. Home care nurses can also contact the memory clinic if they suspect the onset of dementia in their client. The investigation and path to a diagnosis are conducted according to national guidelines [52]. Memory clinics in their current form were initiated in primary care in the late 1990s [53] and the first version of national guidelines for dementia care published in 2005 [52].

The dementia nurse invites the client to participate in an initial assessment of the situation. Testing of cognitive function is conducted using the Mini-Mental State Examination [54] (MMSE) and the Consortium to Establish a Registry for Alzheimer’s Disease [55] (CERAD) test. A standard series of blood tests are conducted as well as a magnetic resonance imaging (MRI) or computerized tomography (CT) scan of the brain. The combined results of the investigation are evaluated by the memory clinic geriatrician who then meets with the patient in order to communicate the diagnosis and possibly prescribe medication. After meeting with the geriatrician, the patient sees the dementia nurse to clarify potential misunderstandings and to go through the information received as required. Follow-up visits at the clinic and in the home are scheduled according to a timetable which varies from municipality to municipality depending on resources available. The mean length of time from problems being noticed to diagnosis, was found to be 2,24 years as noted by a 2018 survey of family carers’ experiences [56], a survey in which Finland was included.

An invitation to participate in the study was sent via e-mail to a total of 29 dementia care professionals within a Finnish wellbeing services county in Western Finland. Seventeen of the invitees participated in the study. In the invitation to participate, introductory questions and themes were listed as outlined in Table 2. Researcher networking was utilized to identify interviewees; therefore, recruitment was purposeful. The participants worked within a primary care area with a wide geographical spread. The interviews were arranged in the participants’ own settings. The inclusion criteria for participants were experience in out-patient dementia care and willingness to participate in the interview. The majority of participants were registered nurses (*n* = 13). The remaining participants consisted of two geriatricians, one social worker, and one professional of applied gerontology. All participants were female and had worked with dementia clients in memory clinics and/or a home care setting. Work experience in dementia care ranged from 20 + years to 1,5 years. Nine of the nurses had continuing education in dementia care.

**Table 2:** Focus group interview introductory questions

*• In what phase of dementia illness should ACP be initiated?*
*• Whose responsibility is it to initiate ACP in dementia care?*
*• What are the ethical aspects in conducting ACP as well as in refraining from doing so?*
*• How could ACP be organized to be beneficial for the person living with dementia and his/her family caregiver?*

### Data collection

Data were collected by two researchers (the first and the second author) in August 2022 via three semi-structured focus group interviews. The focus groups included 4, 5, and 8 participants respectively. The two geriatricians who participated were included in the group with 8 participants. All interviews followed a protocol starting with a description of the PhD project and proceeding to open-ended questions. The first author, who possesses extensive ACP knowledge, functioned as moderator posing questions and moving the discussion forward while the second author observed, took notes, at times asked follow-up questions, and in conclusion presented a summary of the interview. After each session, the second author debriefed with the moderator and gave feedback on the session [47]. Each focus group interview was digitally recorded. Verbatim transcription of the interview recordings was performed by a research assistant. Participants were highly involved in the interview situation and gave a rich account of their views on and experiences with planning for future care with their patients, resulting in sessions that lasted an average of 1 h 5 min and totaled 57 pages of text with a word count of 28,728. Data sources also include the field notes of the second author as well as the post-interview debriefing notes of the first author.

### Data analysis

**Fig. 1:**
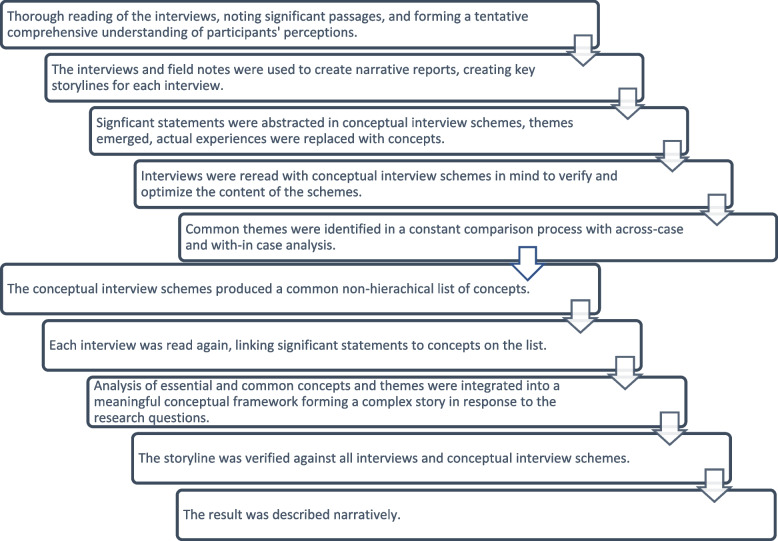
The stages of the data analysis

A modified version of the Qualitative Analysis Guide of Leuven (QUAGOL) [57, 58] was used for the two-step analysis. In this modified version, a software program for analysis was not used. As the interviews were conducted in both Finnish and Swedish, the use of a software program for coding was deemed inappropriate. QUAGOL provides a systematic, comprehensive, and multifaceted approach to the analysis of complex qualitative data. A case-oriented narrative approach is combined in a systematic analytical process [58]. Figure 1 describes the interconnected stages of the analysis. All authors participated in the data analysis, continuously discussing emerging results.

An example of a narrative report can be found in Supplemental file 1. An example of the analysis path for sub-theme 1 is presented in Table 3.

**Table 3:** An example of the analysis path

Significant statements	Key elements	Sub-theme
Stage 3 on the Global Deterioration Scale/Functional Assessment Staging latest, it’s been too late for care planning All clients don’t understand the diagnosis and what it means During subsequent home visits by the nurse, the progression can sometimes be talked about There are clients who don’t understand that they have a dementia illness, they lack illness insight and even deny illness When clients come in for assessment, they’re already in stage 3 or 4 At stage 4 you no longer have the capacity to make your own decisions Such clients don’t want to think about the future or a living will as they perceive themselves to be young and healthy Clients ought to come for assessment in an earlier stage When the client comes for assessment the illness can have progressed far Not everyone is interested in an assessment Barriers to contact the clinic can be challenging Sometimes the client’s illness has progressed far Single clients often come to assessment late in the trajectory	Delayed diagnosis	The person with dementia: losing capacity for self-determination, losing oneself

### Ethical considerations

The ethical principles of The Finnish Advisory Board on Research Integrity have guided the current study [59]. The study was granted research permission by the Board for Research Ethics (FEN) at Åbo Akademi University as well as by the Research, development and innovation department of the Wellbeing services county in which the study took place. All participants received both written and oral information about the aim of the study. They were informed of the voluntary nature of participation, that the interviews would be recorded, and that results would be presented on a group level so that individual participants could not be identified. Participants gave their written informed consent before the interviews. The data were stored in password protected computer files. In the transcripts, any personally identifiable information was removed. Only the researcher group had access to the files.

## Results

The data analysis identified one main theme with three sub-themes describing the views of dementia nurses and geriatricians on ACP in dementia care and the factors that affect the ACP process. The main theme is the ‘perfect storm’ caused by multiple forces and circumstances coming together and affecting the ACP process in dementia care. The unfavorable circumstances creating this ‘perfect storm’ consist of the nature of the illness, the demands placed on the care professional, as well as insufficient resources. The sub-themes are:

1. The person with dementia: losing capacity for self-determination, losing oneself with the key elements being delayed diagnosis, client illness trajectory and illness insight.
2. The person with dementia and the care professional: Building a trusting relationship where the dementia care process is the key element
3. The care professional: skimming the surface or diving into the ACP conversation where the professional carer is the key element.

The sub-themes are presented in the form of a narrative storyline.

1. *The person with dementia: losing capacity for self-determination, losing oneself*
  The study participants were concerned with the delay in investigation and late diagnosis of dementia. When people get in touch with the memory clinic due to a concern with their own health or with the health of a loved one, the dementia has often reached a moderate stage.

*They [patients]have a 15 [points] on the MMSE test, I would say, about, and actually, already at the first or second visit, we’ve had to start thinking about a nursing home. And it’s not supposed to be that way. (Focus group 1, (FG1), dementia nurse 1 (DN1)).*

People are also at times reluctant to be assessed and the perceived barrier to the memory clinic can feel challenging. Once the diagnosis is communicated, the person with dementia and their family caregiver (the dyad) might lack understanding for and knowledge about dementia, its consequences, and the illness trajectory. Some people with dementia lack illness insight altogether and even deny being ill. People with dementia react in their own way to the diagnosis. Some people with dementia do not want to talk about the illness or future care at all as the situation can get too emotional and anxiety filled. The diagnosis often comes as a shock.

*We would like them to get in touch earlier so that the dementia diagnosis could be made as early [in the illness trajectory] as possible. But if no one reacts to it nor gets in touch. Or we get a referral, well, we don’t go investigating anyone just like that. (FG1, DN3).*

While the study participants agree ACP should be introduced as soon as possible in the care path, they also state the first time one meets with the person with dementia is not the right time due to the information overload, sensitivity, and sometimes shock of the situation. The first follow-up visit after 4–6 months is seen as a better time to introduce ACP. However, due to care contact often being established relatively late in the illness trajectory, the dementia nurses sometimes experience that it is too late for ACP involving the person with dementia during the follow-up visit.

2) *The person with dementia and the care professional: building a trusting relationship*

While the participants viewed ACP as something that is or ought to be part of the care process, ACP is often a conversation that does not take place, as participants felt it was important to build a trusting relationship before introducing more sensitive topics. As noted in the first theme, time of diagnosis is viewed as the wrong time for discussing future care. There is a great deal of information shared during the diagnosis visit with the geriatrician. After seeing the geriatrician, the dyad meet with the dementia nurse who tries to ensure the dyad has understood the diagnosis, discusses medication if it has been prescribed, and informs the dyad about the future care pathway. At this point, illness trajectory, prognosis, living wills, and lasting power of attorney are not discussed unless the person with dementia and/or their family caregiver initiates such discussions.

*It's good to do [ACP] in the early phase of the illness, when you find it, so that it won’t be too late, which it often gets to be, but you can’t do it the first time you meet a patient, not even when the diagnosis has been communicated at an earlier visit [by someone else]. You have to create trust and a rapport with the patient before you can start talking about these kinds of difficult things, and that’s why it often gets pushed to a later stage. (FG2, geriatrician 1(G1)).*

During the first or second follow-up visit at the home of the person with dementia, a lasting power of attorney form is often introduced along with a form for a living will. There are several different forms for living wills in circulation in Finland and the nurses have specific ones they prefer to use.

*Yes, sometimes we just give [the form] to them, sometimes they are the kind that don’t really want to, they don’t even want to take it with them, and sometimes we review it together, the city of Sibbo and the Alzheimer’s Society have co-created the living will form, and it’s the kind I like to use, it’s clear, it tells a bit more about such things. (FG3, DN2).*

However, the living will form is not always reviewed and completed together with the person with dementia unless he/she requests it. The person with dementia is asked to fill out the form by them self with the help of family and to turn it in during their next visit or register the form at the general health clinic. In the patient journal systems used, there is no easy way to document the wishes of the person with dementia in a clear manner which is quickly visible to other professional care staff.

*Well, I always think that sometimes, well, these forms they are of course different, but sometimes there are such medical words and terms, that an ordinary person, an ordinary person doesn’t know, what it’s like, what it’s like to have a peg [percutaneous endoscopic gastrostomy] (FG3, DN4).*

The national guidelines for dementia care provide a clear, generic path for dementia nurses and geriatricians but no detailed checklist, tool, recommendations, or model for ACP. The majority of participants stated that a checklist would not work as each person with dementia is an individual and every care situation unique. However, some form of structure or support was viewed as having the potential to be useful, especially for new dementia nurses.

*It would be pretty difficult to have a certain kind of conversation model… it’s little by little… (FG2, DN4).*

*Yes, when everyone is so… it’s so individual… but of course conversation technique is very important, in my experience, so if it doesn’t come naturally, it’s probably good to take some classes. (FG2, DN3).*

Making ACP into a natural and routine part of the dementia care path was seen as an essential facilitating factor. The current process limits number of visits with a doctor or geriatrician to one or two visits total which means that the responsibility for ACP conversations falls to the dementia nurse who may follow-up, care for, and support the person with dementia and their family for many years.

Lack of time and resources were often highlighted as barriers to introducing ACP. The dementia nurses mentioned having too many clients which prevented them from visiting their clients more often and reduced time for deeper conversations. Time for reflection in an undisturbed environment and the support of co-workers were seen as important in facilitating ACP conversations. A trusting relationship and the time to build such a rapport were seen as necessary prerequisites for introducing conversations about sensitive issues such as illness trajectory, lost function, and end-of-life care.

*Well, anyway, I think that one has to get quiet time at work, it is very stressful to have difficult things, if you at the same time feel that you don’t really have the time, because it gets hard for oneself, we’re only human. One needs to talk to one’s colleges, do a bit of briefing… and then some processing in your own head. (FG1, DN1).*

3) *The care professional: skimming the surface or diving into the ACP conversation*

The study participants spoke of intuition around and sensitivity to the emotions of the person with dementia and their family caregivers when approaching sensitive issues. Intuition was described as a type of undervalued silent knowledge acquired through work and crisis handling experience. However, intuition was also compared and contrasted to evidence-based practice and mentioned as something decisions could not be based solely upon. The participants spoke of their own feelings as something which could potentially lead to conscious or unconscious avoidance of difficult conversation topics when caring for people with dementia.

*I’m thinking that one uses feeling and intuition a lot. And with more work experience and life experience, it awakens, wakes up, the intuition grows, so that you can better choose to do or not to do. But in some ways, it’s then on pretty thin ice, when it’s like this, yes… what is it that makes us avoid this kind of conversation, is it our own baggage, our own fear, yes… it’s really a difficult question, when do you cause more good and when more harm, that’s the question we need to activate more. (FG2, G1).*

When reflecting on the challenges of ACP in dementia care, the consensus was that dementia is different than other illnesses. According to the participants’ experiences, there is still a stigma attached to dementia and the associated loss of cognitive abilities. ACP and illness trajectory was viewed as easier to discuss in cases of, for example, incurable cancer where there is a more predictable illness trajectory and time frame. A person can live with dementia for many years which contributes to people with dementia occasionally feeling that planning for future care is not an immediate concern.

*Is it in sense then a societal challenge, this illness? (Moderator).*

*Yes, very much so, there’s still that shame, it’s still like that, [the illness], it’s not understood… (FG3, DN1).*

The insecurity felt by the participants is related to role confusion, to the lack of knowledge about ACP, and to the lack of support and the lack of a strong foundation to stand on for ACP. There was some confusion when it came to the roles and responsibilities of the dementia nurse versus the geriatrician. Dementia nurses felt the doctor at times pushed the responsibility of explaining the diagnosis onto the nurse as well as failed to properly inform the dyad about the life-limiting nature of dementia.

*I think that it’s good that the doctor introduces [a living will] in that stage when the diagnosis comes, the doctor is in a way the authority so that perhaps in that stage a memory of it remains, that this is something we have talked about and that the nurse gives the brochures and the doctor, they don’t need to review it, but the nurse goes through it. (FG3, N2).*

Some of the nurses drew a distinct line between formal decisions and treatment restrictions made and documented by the doctor, the living will document completed by the person with dementia, and the more informal discussions about wishes for future care conducted by the dementia nurses. The nurses also felt they did not have enough training to manage dementia patients in acute psychological crisis caused by being diagnosed with dementia. While having identified critical moments, such as hospitalization or a move to an assisted-living facility, as opportunities to initiate ACP, they wished for more support and training in general on how to approach and introduce ACP and when to do so, especially when caring for a patient who is reluctant to talk about the future. A fear of saddening their patients with ACP conversations at times contributed to the insecurity felt.

*It’s not so difficult to talk about, no, I don’t feel that it’s hard to talk about, but at what point, sort of… (FG3, DN3) Right, and then you still have to be… even though you can manage talking about it, you always have to sort of try to suss out the situation, when [to talk] (FG3, DN2).*

*I don’t feel that I have [tools for ACP], but I really would like to have some, I have the facts knowledge but precisely that, how to approach and how to raise the issue, and [to know] what do I say now… and how to get the other one to listen, the message, when the person is not very receptive, but needs to be… (FG3, DN4).*

While generally acknowledging the positive aspects of ACP in dementia care, the participants emphasized a strong focus on positivity, the maintenance of hope, and support for a good quality of life for people with dementia and their family caregivers. Some of the study participants expressed the view that ACP conversations could potentially remove hope and throw a person with dementia into despair.

*Well, I think it’s good there after 6 months, to discuss and give, bring up both [lasting power of attorney and living will], but to start discussing the end of life then, it’s of course… we’re still supposed to give them hope and try to strengthen them and their self-esteem, a lot of it is based on their self-esteem, that they can actually think that they can manage and feel well. So, it would actually be like pulling the rug from under them. (FG2, DN4).*

The study participants wanted to strengthen the self-esteem of their clients and focus on the possibilities of maintaining hope and a good quality of life despite the illness. Speaking about the end of life and living wills too soon felt wrong to the majority of participants as they felt such conversations would lower the quality of life of their patients. However, study participants vacillated on their views at times, stating that a living will can be a positive thing as well and can be completed in a positive manner. A majority of the participants expressed the view that a living will and lasting power of attorney should be a natural part of every person’s life and something that everyone, whether ill or not, should complete and document, preferably before reaching old age.

The participants reflected on the self as an instrument and tool when caring for people with dementia and their family caregivers. Strong and sometimes difficult emotions arise in dementia nurses and geriatricians when working in challenging situations and with people who are in shock, at times depressed, and/or in denial. Difficult situations also arise in the later stages of illness when the family caregiver may express different wishes for care than the person with dementia has previously expressed. Participants saw it as necessary to have worked through one’s own issues and fear of death to be able to talk about death with their patients. The nurses and geriatricians felt the need to be grounded in themselves to find the courage to initiate such conversations. If a nurse or geriatrician is not grounded in this sense, the choice to steer away from such talks is easier made and the topic often avoided altogether.

## Discussion

The purpose of the study was to investigate the view of dementia nurses and geriatricians on ACP in dementia care. The results of the current study demonstrated that dementia nurses and geriatricians express a generally positive view of ACP in dementia care, but simultaneously hold a number of views about factors which affect the conditions for conducting ACP. The questions of timing and responsibility remain unclear.

The ACP conversation is hampered by people and family caregivers contacting the memory clinic late in the illness trajectory which may give the dementia nurse and the geriatrician the perception that it is already too late to introduce planning for end-of-life care. In a 2018 survey of family carers’ experiences in five European countries [56] in which Finland was included, a high prevalence of the person with dementia refusing to seek help was reported by 46,3% of carer respondents. Another cause for the delay was the first professional seen not considering anything to be wrong, as reported by 26,3% of respondents. Referrals to diagnostic services taking a long time were reported by 15,4%. The view that it is already too late to introduce ACP is deepened by the long intervals between follow-up visits. In our study dementia nurses and physicians also reported a lack of knowledge among people with dementia and their family caregivers about illness trajectory, prognosis, and dementia in general. These views are supported by the aforementioned survey [56] and other studies as well [60, 61]. Moore, Goodison, and Sampson noted in a 2018 study [30] that dementia clinics have mixed views about the appropriateness of disclosing the terminal nature of dementia to people with dementia. The mixed emotions about informing people with dementia and their family caregivers about potential illness progression has been well documented [6] and was noted in the current study.

As ACP has not received much attention in everyday healthcare work in Finland [37, 39], there is a general lack of knowledge of the ACP process and an ACP conversation is often reduced to the completion of Advance Directives through different forms provided by the Alzheimer’s Society, for example. There is a lack of distinct guidelines and tools for ACP in dementia care adapted to the Finnish care context which further challenges the initiation of ACP conversations. In Finland in general, documentation of healthcare and care decisions are fragmented in many different journal systems which do not communicate with each other, making the identification of critical decisions difficult. Dementia nurses and physicians report a shortage of staff which increases the number of clients to dementia nurse as well as the amount of time between follow-up visits. As allotted time per person with dementia becomes increasingly pressured, ACP conversations tend be left undone or to take a back seat to matters that are considered more urgent, such as medication reviews and the challenges of living at home with a caregiver spouse.

Dementia nurses and physicians indicate that an undisturbed environment and time are factors that facilitate discussions about and planning for future care. Time with individual clients would increase if the client to nurse ratio was increased so that each nurse would have fewer clients to follow-up and attend to. Developing and building relationships with clients in dementia care was seen as key in enabling ACP discussions, a key factor noted in other studies as well [19]. Dementia nurses also stated that support from the physician is often necessary and indeed mandatory when it comes to decisions such as palliative care decisions or medication decisions. While the dementia nurses feel knowledgeable about forms relating to Advance Directives, illness trajectory, and the care path recommended by national dementia care guidelines, it was noted that more distinct ACP guidelines and clearer role distribution between nurses and physicians would facilitate ACP in dementia care. The detailed checklist approach is not seen as helpful, which is a notion supported by a 2017 editorial discussing ACP and Advance Care Directives in which Komesaroff states what is needed is not complicated and refined protocols and checklists, but a “continuing awareness of the key role of open ethical dialogue in the practice of all aspects of clinical care” [62].

Trying to normalize ACP conversations and making them part of routine care as well as using critical moments or key triggers to initiate the ACP process are seen as other facilitators. Critical moments can include the termination of mitigating medication, the need for home care services, or contemplating moving to a nursing home [17, 21]. In our study, dementia nurses note that in caring professions, one uses the self as a tool in the care situation and process. Using one’s intuition honed through many years of working with dementia patients and their family caregivers is seen as a strength in the balancing act between enabling hope and maintaining realistic expectations.

Dementia nurses and geriatricians highlight wanting to do good and to act in the best interests of the patient. Dementia nurses and geriatricians state they know what ought to be done, but planning for future care with their patients and family caregivers does not at times take place. To talk about death and end-of-life care is seen as potentially anxiety-inducing and the thought of one’s own death avoided. Not having come to terms with one’s own mortality and lacking the time and the support to dive into challenging conversations, as well as diverting from the sorrow, grief, and anxiety of the person with dementia by focusing on a positive attitude contribute to the absence of ACP or, in a sense, to “skimming the surface” of the ACP process. An important ethical question to address is: can hope and ACP exist side by side? The feeling that discussing the progressive and terminal nature of dementia is contradictory to focusing on living well with dementia has been found in other studies [30, 63]. A recent meta-review identifying moral barriers and facilitators encountered by physicians in ACP discussions with people with dementia described moral dilemmas that can lead to avoidant behavior concerning ACP [64]. The burden of a high patient to nurse ratio reduces time available with each patient and the weariness of a heavy workload contributes to an avoidance of sensitive and emotionally challenging subjects. The dementia nurses in the current study felt a need to function as advocates and to protect their patients from feelings of hopelessness and anxiety, similar to nurses in an oncological context [65].

In the Finnish national guidelines for post-diagnosis dementia care, some of the best care practice steps are listed as follows: 1) the dementia diagnosis should be explained to both PWD and family caregiver, 2) a care plan should be made after diagnosis, 3) symptom-based medication for progressive dementia needs follow-up, 4) expertise is needed for anticipation and treatment of behavioral symptoms, 5) there needs to be a holistic approach to the general health of the person with dementia, including an assessment of nutritional status, 6) the following documents should be part of the care; guardianship and lasting power of attorney as well as a living will [52]. The national guidelines give no further instructions on when, how, and who should conduct ACP. The living will discussion is often the part omitted from the care process for reasons discussed in previous paragraphs. It can be argued that omitting the living will part of the care path is a form of missed care. Studies show that ACP interventions for people with dementia can have positive effects and have the potential to provide a sense of relief [16]. For family carers, ACP for older people can have a stress, anxiety, and depression reducing effect [66].

Any aspect of nursing care that is delayed or altogether omitted in whole or in part is referred to as missed care or care left undone [67]. According to Suhonen and Scott (2018), missed care can be seen as “an outcome of activities and processes performed (or not performed), consciously or unconsciously, by professional nurses” [68]. Inadequate time, skill mix, and staffing level contribute to the failure to carry out or withholding of necessary nursing tasks [69]. The topic of missed care has been studied most in acute care hospitals but has been found to be a common issue in nursing contexts [68]. A recent study focusing on missed care in community and primary care settings found that there is a high prevalence of understaffing in community nursing, making missed care more likely to occur [70]. Suhonen and Scott (2018) suggest considering the ethical basis for resource allocation and highlight resource constraints on available nursing time as a necessary and urgent public, national and international discussion [68].

The argument that everyone should have a living will no matter what health issues and diagnoses has been suggested in other studies as well [71]. How to educate the general public about dementia, lasting power of attorney, and living wills, in short Advance Care Planning, remains a complex subject to be further studied.

### Strengths and limitations

The preparation, data collection, and analysis stages were documented and performed with care to enhance the trustworthiness of the research. The Standards for Reporting Qualitative Research (SRQR) [50] were followed. The sampling method chosen ensured that knowledgeable participants were recruited. The study participants were engaged in the focus groups interviews, producing a large amount of meaningful data. The knowledge and experience of the study’s participants provided a diverse and deep understanding of the research phenomena which has previously not been extensively explored in the Finnish context. These findings provide important information for the development of ACP in dementia care in this context as well as in other regions with similar dementia care processes. The first author’s knowledge about ACP guidelines, jurisdiction, and terminology may be considered both a strength and a weakness of the study. There might be a risk of “blindness” to certain aspects of ACP. However, ongoing reflection and sensitivity to the material, the participation of the second author in the focus group interview sessions, and all authors participating in the data analysis provided opportunities to consider any presuppositions during the interviews and analysis stages. Limitations associated with small data samples are present in the current study as well, including limited generalizability. Therefore, the main findings need to be further explored in related settings.

There are possible stumbling blocks connected to the QUAGOL analysis method [57, 58], including losing track of the research question, information overload, and focusing on creativity and intuition. The use of field notes and the documentation of reflections in the analysis process have enhanced trustworthiness. Credibility was affirmed by continuous research team discussions about the emergent results.

## Conclusion

In our study, dementia nurses and geriatricians acknowledge the importance of advance directives and hold a generally positive view of ACP in dementia care. A number of factors affect the conditions for conducting ACP. The lack of ACP in dementia care can be seen as a form of missed care caused by multiple forces coming together simultaneously. The unfavorable circumstances creating this ‘perfect storm’ consist of the nature of the illness and the associated stigma, unclarity in the suggested care path with inadequate guidelines for ACP, the demands placed on dementia nurses and geriatricians, as well as insufficient resources. Creating a trusting and caring relationship, getting to know the person with dementia and their family, using intuition and sensitivity in the timing of the ACP conversation, as well as receiving support in the form of coaching and time to reflect with co-workers are all prerequisites for a relationship-centered ACP process, a process with the potential to be rewarding for both care professionals and the person with dementia and their family caregiver.

## Supplementary Information

**Additional file 1: ** Focus group interview 1 Narrative report (page 1 of 4).

## Data Availability

The data generated and analyzed during the current study are not available for public use, due to confidentiality, but are available from the corresponding author on reasonable request.

## Abbreviations

ACP: Advance Care Planning
CERAD: Consortium to Establish a Registry for Alzheimer’s Disease
CT: Computerized Tomography
DN: Dementia Nurse
FG: Focus Group
G: Geriatrician
MMSE: Mini-Mental State Examination
MRI: Magnetic Resonance Imaging
QUAGOL: Qualitative Analysis Guide of Leuven
PhD: Doctor of Philosophy
RN: Registered Nurse
SRQR: Standards for Reporting Qualitative Research
