# Supplementary material for: 'A perfect storm' or missed care? Focus group interviews with dementia care professionals on Advance Care Planning
Source: BMC Geriatr. 2023 May 21;23:313. doi: 10.1186/s12877-023-04033-7 (PMC10200042; doi:10.1186/s12877-023-04033-7)
Supplement: Supplementary file 1 — Additional file 1: Focus group interview 1 Narrative report (page 1 of 4). [file 12877_2023_4033_MOESM1_ESM.docx]

**Supplemental file 1.**

**Focus group interview 1 Narrative report (page 1 of 4)**

The interview starts with a question about timing when it comes to conversations about ACP. One of the nurses right away refers to GDS-7 with the 7 stages of dementia. The scale is used fairly often in dementia care, the nurse gets a picture of what phase the client is in and it is also used as **a tool for explaining and describing for the client and family** so that they can get an understanding as well for what phase the client is in. The nurse says that at **stage 3, it has been too late for ACP** or a living will, **at stage 4 it is too late**. At stage 4 one is **no longer capable** of making good decisions on one’s own, according to the nurse. When clients come in for an assessment, they are often **already in stage 3 or 4** with an MMSE on about 15. Already during the first or second visit, one has had to start contemplating assisted living or a nursing home. The nurses would like the clients to come **sooner for assessment**.

To **make a living will before one gets** sick is mentioned as a good thing. But it is also noted that as life goes on, **one can change one’s mind** or forget what was wished for and wanted. A nurse states that with the ones who come in time (for assessment), the nurse can talk about ACP with but **not during the first visit**. There is **so much information** to take in during the first visit. **Trust is needed** and you start to **build a relationship** with the client and his/her family which is necessary for ACP. **Sensitivity and trust** is needed. It’s not possible to discuss serious life-changing matters with someone you don’t know. **It doesn’t feel like the right moment to talk about the end when you are at the point of communicating the diagnosis**. To **facilitate giving information** about possible illness development, the GDS-7 is used and is perused with the client. The classification of the stages is helpful but the nurse **doesn’t want to approach the topic of how it all will end.**

The client meets the **doctor who communicates the diagnosis** and then the client meets with the nurse. All clients **don’t understand the diagnosis** and what it means. The doctor has a tendency to talk about it lightly and in a way **push the diagnosis explanation on to the nurses**. Some clients are very **saddened** by the diagnosis. **The doctor doesn’t talk with the client and the family about dementia being an illness leading to death**. During subsequent home visits by the nurse, the **progression** can sometimes be talked about. A living will form is **not brought up during the first home visit** but maybe during the second. The living will form on Kanta (national patient data repository) is sometimes used. Before moving to an assisted living facility, a type of **life narrative** is used where advance directives can be brought up. There are clients who **don’t understand** that they have a dementia illness, they **lack illness insight** and even **deny illness**. Such clients don’t want to think about the future or a living will as they perceive themselves to be young and healthy.
